# Learning to adaptively cooperate through social interactions during childhood and adolescence

**DOI:** 10.1038/s41539-026-00423-9

**Published:** 2026-04-04

**Authors:** Rong Zhang, Lei Zhang, Xin-Min Hao, Jean-Claude Dreher, Chen Qu

**Affiliations:** 1 School of Psychology, South China Normal University, Guangzhou, China; 2 Centre for Human Brain Health, University of Birmingham, Birmingham, UK; 3 School of Psychology, University of Birmingham, Birmingham, UK; 4 Institute for Mental Health, University of Birmingham, Birmingham, UK; 5 Centre for Developmental Sciences, University of Birmingham, Birmingham, UK; 6 Laboratory of Neuroeconomics, Institut des Sciences Cognitives Marc Jeannerod, CNRS, Université Claude Bernard, Lyon, France

**Keywords:** Evolution, Neuroscience, Psychology, Psychology

## Abstract

Humans develop the ability to navigate social environments by recognizing others’ behavioral patterns, which evolve across development. We asked how children adapt their learning strategies to opponents in a social dilemma (Chicken game), focusing on trial-by-trial, opponent-sensitive adaptation. Study 1 included preschoolers, while Study 2 examined school-age children and adolescents. Across ages, children adjusted their choices to their opponents, showing more cooperative behavior (“Swerve” choices) against high-competitive opponents and more competitive behavior (“Go straight” choices) against low-competitive opponents. Reinforcement learning model comparison suggested age-related changes in the relative support for reward-based versus belief-based accounts. These findings provide computational evidence consistent with developmental changes in how children adapt to competitive versus cooperative partners.

## Introduction

In real-world social interactions, humans continuously learn about the traits, trustworthiness, and preferences of others through social exchanges, adjusting their own actions accordingly^1–3^. For example, in contexts where cooperation and competition coexist, individuals may strategically choose to cooperate when others compete, or compete when others cooperate^4–7^. Even young children demonstrate the ability to selectively share resources or adaptively choose partners^8,9^, showcasing early competence in navigating these complex dynamics. Reinforcement learning (RL) models and their extensions (e.g., fictitious play, influence RL models) provide a powerful mathematical framework to explain how individuals adapt their learning during social interactions, which subsequently shapes their decisions and actions^10–15^. Research has shown that adults can flexibly learn the beliefs of others to guide their social actions, employing higher-level learning strategies^16–18^. Recent advances suggest that individuals begin learning to understand others’ preferences from early childhood, using an intuitive, generative model based on observed evidence^19–21^. However, a critical question remains: when and how do children develop the ability to distinguish behavioral tendencies and navigate these interactions effectively? What are the learning strategies during different developmental stages? To address these questions, we compared reward-based updating with belief-based tracking of opponent tendencies using Bayesian hierarchical RL models, investigating the adaptive nature of social learning from childhood to adolescence.

Over the past decade, RL models have been extensively used to study human decision-making in both social and non-social contexts^13,22–25^. For example, Q-learning models propose that individuals incrementally update their estimates of action values based on prediction errors, scaled by their learning rates. In non-social contexts, adults typically show higher learning rates for negative outcomes and demonstrate greater flexibility in adjusting their strategies across contexts^26,27^. In contrast, younger children tend to exhibit higher learning rates for positive outcomes, potentially due to optimism bias, while learning rates for negative outcomes gradually increase from childhood to adolescence^28,29^. During social interactions, the actions of others serve as effective rewards (e.g., sharing or not), enabling individuals to update the expected value of their own behavior. For instance, participants update trustworthiness or closeness through social prediction error^30^, learn social affiliation from intention and outcome feedback^31^, and select cooperative partners for themselves and others^32^.

Social dilemmas reflect real-world challenges involving human cooperation and conflicts of interest between self and others. Economics games such as the Public Goods Game, Prisoner Dilemma, and Chicken Game provide valuable insights into how individuals interact with others^33,34^. Individuals can develop dominant strategies over repeated interactions with opponents who have fixed preferences, such as cooperating with a competitive opponent to avoid potential losses^1,2^. By capturing current rewards, adults can quickly learn opponent’s behaviors through basic RL to maximize benefits and adapt strategies flexibly^3^. The ability to apply optimal social learning strategies is critical for effective interactions in social dilemmas, as it depends on factors such as latent rewards, social context (e.g., cooperation or competition), and beliefs about the interaction partners.

While a simple reward-based RL model may not fully account for strategic learning, belief-based learning involves inferring and updating beliefs about others’ future actions by analyzing behavioral history and internal states, such as intentions or beliefs, enabling more adaptive decision-making^15,16,21,35^. Research has shown that in competitive games, adults employ higher-order strategies to infer opponents’ mental states and optimize their outcomes^17,36^, exhibiting asymmetric learning processes when interacting with different types of opponents^4,37^. However, little is known about the computational mechanisms underlying how children learn others’ behavioral patterns.

Humans engage in social interactions from infancy, gradually developing adaptive behavior that goes beyond simple imitation. Children around 4-years old show flexibility in different social contexts by following game rules, adapting to a trucking game by exhibiting affiliative behavior in cooperative contexts and antagonistic behavior in competitive ones^38^. Around ages 5 to 6, children begin to cooperate, showing increasing prosocial or cooperative behavior as they grow^39–44^. In the Chicken game, recent studies have found that preschoolers, around 5 years old, can learn their partners’ behavioral tendencies and begin to make coordinated decisions to maximize shared rewards^45^. Older children, between 6 and 9 years old, increasingly regulate their behavior and coordinate with others by applying rules or strategies^46,47^. In adolescence, social learning is particularly responsive to peer influences, both positive and negative^24,48^. However, these studies mainly relied on real-life interaction games without strict experimental control, making it difficult to track the computations underlying children’s learning processes. To address which computations best account for the development of the ability to learn others’ behavioral tendencies and navigate social interactions, we adapted a Chicken game to children and adolescents. This enabled us to precisely manipulate opponents’ behavior and to apply RL models to assess learning mechanisms. This approach also allowed us to test whether age-related differences in model support would emerge across development. In particular, we examined whether children’s choices increasingly reflected tracking of opponent regularities with age. From a cognitive-developmental perspective, this potential shift aligns with the observation that younger children tend to rely more on outcome-driven (model-free) learning, while with development, they place increasing weight on the tracking of behavioral regularities (model-based), a process supported by the growth of more abstract representations of rules and patterns^38,49^. As for adolescents, their heightened sensitivity to peer influence and social rewards further motivates the use of these sophisticated, belief-based learning strategies^24^. We also considered that belief-based strategies may recruit advanced cognitive abilities, such as Theory of Mind (TOM), which develops across childhood and may constrain children’s ability to infer others’ mental states^50–53^. This raises a fundamental question in children’s social learning: do younger children rely more on reward-based learning, with older children showing greater support for belief-based accounts?

The present study aimed to investigate social learning behavior from early childhood to adolescence, examining whether children rely more on reward-based or belief-based learning when interacting with different opponents and how this process evolves across development. To this end, children from preschool, primary school (grades 1–2 and grades 4–5), middle school (grades 7–9) and high school (grades 10–12) completed a child-friendly version of the Chicken game. All children interacted with two types of opponents, high-competitive opponents (HCO) and low-competitive opponents (LCO), which were manipulated with more aggressive and more submissive strategies. Previous research indicated that 5-year-old children can independently coordinate with others, whereas 3-year-olds struggle to understand such tasks^42^. We therefore began with 5-year-old preschool children. To ensure age-appropriate comprehension, Study 1 used a simplified payoff matrix for preschoolers (without negative losses), whereas Study 2 (6–17 years) used a full matrix suitable for older children and adolescents.

At the behavioral level, we hypothesized that children would adapt their choices based on the type of opponent, exhibiting higher cooperation rates with competitive opponents and lower cooperation rates with cooperative opponents. As for the computational results, we applied RL models using a Bayesian hierarchical approach to fit the data and examine how learning strategies (reward-based vs. belief-based learning) evolve across development. Model comparisons helped identify the best-fitting model explaining the underlying psychological mechanisms. We expected younger children (e.g., preschoolers) to rely on reward-based learning, whereas older children (e.g., adolescents) would incorporate opponents’ behavioral tendencies. We also explored whether children’s learning rates varied depending on the type of social interaction.

## Results

### Preschoolers gradually learn to cooperate more with competitive than with cooperative opponents (Study 1)

Preschool children gradually learned to differentiate between competitive and cooperative opponents over repeated interactions. We computed the cooperation rate (choosing to Swerve) for each block of 10 trials as the dependent variable and ran a repeated-measures ANOVA with Opponent (HCO vs. LCO) and Block (four blocks) as within-subject factors. Because Mendoza’s test of sphericity was significant, we applied the Greenhouse–Geisser correction, which resulted in fractional degrees of freedom.

We found a significant Opponent × Block interaction, *F* (2.29, 117.14) = 4.00, *p* = 0.016, ${\eta }_{p}^{2}$ = 0.07, indicating that preschoolers’ cooperation depended jointly on opponent type and learning over time. Specifically, children showed higher cooperation rates when facing HCO compared to LCO during block 3 (*d* = −0.39, SE = 0.02, *p* = 0.014) and block 4 (*d* = −0.46, SE = 0.02, *p* < 0.001), while there were no significant differences between block 1 and 2 (*p* = 1). Moreover, when interacting with HCO, children cooperated more in block 4 than in block 1 (*d* = −0.34, SE = 0.02, *p* = 0.012), whereas cooperation rates did not differ across blocks when interacting with LCO. We also observed a significant main effect of opponents, *F* (1, 153) = 11.77, *p* = 0.001, ${\eta }_{p}^{2}$ = 0.18, indicating that participants exhibited higher cooperation rates when facing HCO than LCO (*d* = −0.24, SE = 0.01, *p* < 0.001). There was no main effect of Block, indicating that average cooperation did not change across blocks irrespective of opponent type (*p* = 0.277).

### Preschoolers’ choices are best explained by reward-based learning with higher learning rates to competitive opponents (Study 1)

**Fig. 1:**
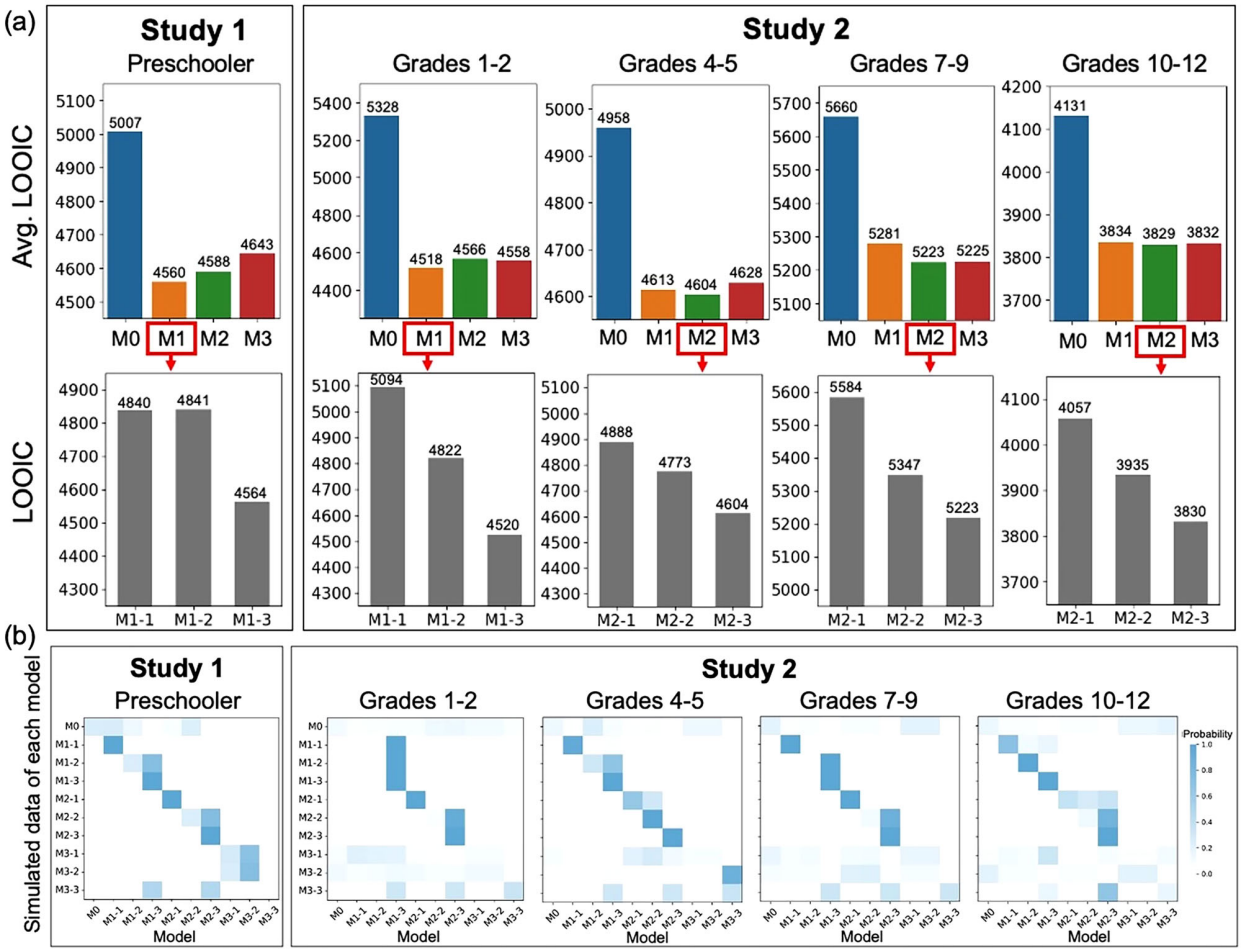
Model comparison and model recovery. **a** LOOIC values were calculated for model comparison, Bayesian model averaging across its three sub-models. M1 (reward-based model), M2 (belief-based model), and M3 (second-order belief-based model) outperformed M0 (non-learning model) across all age groups. In Study 1, preschoolers showed greater relative support for M1, and in Study 2, the same pattern was observed in grades 1–2. By contrast, grades 4–5, 7–9, and 10–12 showed greater relative support for M2. Within the more strongly supported model family, the third variant consistently showed the best fit, with M1-3 receiving the strongest support in younger children and M2-3 in older children. **b** Model recovery analysis. Each model was used to generate synthetic datasets, for each of which model fitting and comparison were performed. Each row is for one simulated model. Each column is for one fitting model. The color in each cell codes the probability that the synthetic datasets from the simulated model in the column are best fit by the fitting model in the column, with a darker color indicating a higher probability.

Computational modeling showed that preschoolers’ trial-by-trial choices were best captured by a simple reward-based learning strategy that differentially tracked competitive versus cooperative opponents. All models converged well, with $\hat{R}$ values for all parameters being less than 1.1. The LOOIC values for all models are provided in the Supplementary Table S1. The LOOIC results indicated that all other models performed better than non-learning model M0, and models that included the parameter $\theta$ performed better than those without, suggesting that participants had a clear behavioral preference in the current task (Fig. 1a). To ensure that preference alone did not account for most of the variance in the models, we conducted a preference-only model (M4). The results showed that models including both preference and learning performed better than the preference-only model. Further model comparison revealed that M1-3 was the best-fitting model (LOOIC = 4564.48), which posited that children’s cooperative behaviors were based on the current reward, following a simple RL rule, and that children had different learning rates for different opponents. Posterior predictive checks (PPC) showed that M1-3 (${r}_{{LCO}}$ = 0.86, ${r}_{{HCO}}$ = 0.85) had a high correlation with the actual behavioral data at the trial-level (Supplementary Fig. S4). A model recovery analysis further confirmed that the best performance of M1-3 was real and could not be attributed to model misidentification (Fig. 1b).

**Fig. 2:**
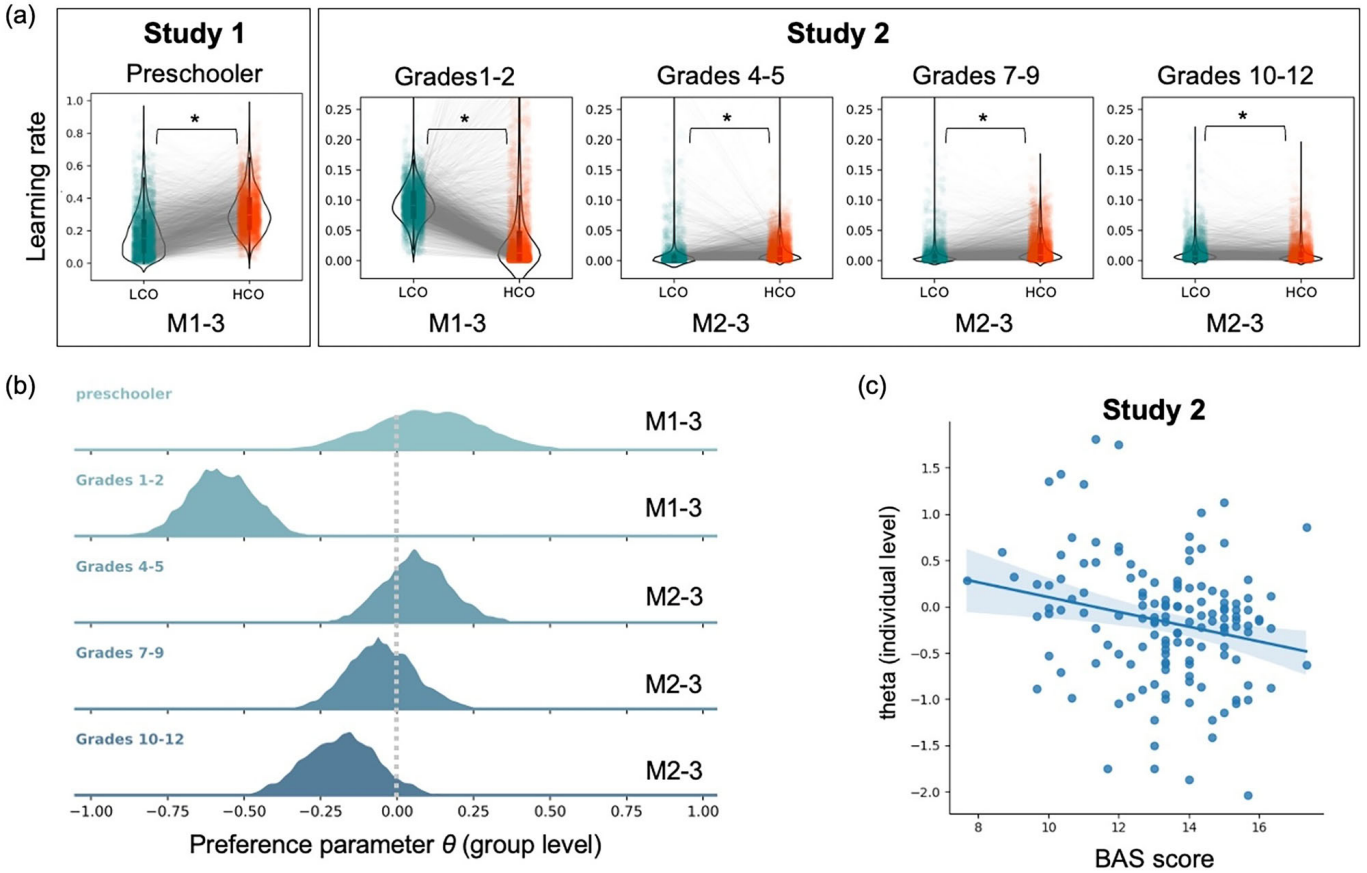
Model parameters. **a** Learning rates of the best fitting model across all grades. M1-3 show that preschoolers in Study 1 have higher learning rates for HCO than LCO (BF_10_ > 100), whereas grades 1–2 in Study 2 show higher learning rates for LCO than HCO (BF_10_ > 100). M2-3 indicate that grades 4–5 and grades 7–9 have higher learning rates for HCO, while grades 10–12 have higher learning rates for LCO. **b** The posterior distribution of preference parameter at group-level was extracted from all best-fitting models, representing behavioral preferences during the task. In Study 1, preschoolers showed a slight preference for choosing *Swerve*. In Study 2, children in grades 1–2 exhibited a strong preference for *Go straight*, while those in grades 4–5 showed an obvious preference for *Swerve*. However, this preference gradually declined with age. **c** shows the BAS scores negatively correlated with the individual-level mean of $\theta$.

We next examined the group-level hyperparameters of M1-3 (Tabel S6). Preschoolers showed higher learning rates when interacting with HCO (*M* = 0.32, SD = 0.11, 95% HDI = [0.105, 0.561]) than with LCO (*M* = 0.20, SD = 0.15, 95% HDI = [0.2 × 10^−4^, 0.519]). A Bayesian Wilcoxon Signed-Rank Test showed a significant difference between ${\alpha }_{h}$ and ${\alpha }_{l}$, BF₁₀ > 100, *W* = 1.26 × 10^6^, indicating that children updated more strongly from outcomes when facing competitive opponents. We also compared preference parameter $\theta$ with 0 using a one-sample Bayesian t-test, finding that children had preferences for choosing *Swerve*, BF₊₀ > 100, *M* = 0.09, SD = 0.19, 95% HDI = [−0.284, 0.476] (Fig. 2b). We also found that preschool children had lower temperature parameter (*M* = 0.37, SD = 0.15, 95% HDI = [0.095, 0.687]), indicating that they exhibited more exploration in their choices.

At the individual level, we used a linear regression model to assess the predictive utility of the preference parameter $\theta$. The model showed that $\theta$ strongly predicted average cooperation rates, *B* = 0.17, *R*^*2*^ = 0.91, *p* < 0.001, 95% CI = [0.159, 0.189], which means $\theta$ could represent individual preferences of choice. Furthermore, a bivariate Pearson correlation analysis revealed a significant negative correlation between language development and θ (*r* = −0.3, *p* = 02), suggesting that children with more advanced language skills were more likely to adopt competitive (Go straight) choices.

### Cooperation peaks in late childhood while children across school age learn to distinguish competitive from cooperative opponents (Study 2)

Across school age, children reliably learned to distinguish competitive from cooperative opponents, with overall cooperation peaking in late childhood. We again used the cooperation rate (Swerve) per block as the dependent variable and ran an ANOVA with Opponent (HCO vs. LCO) and Block (five blocks) as within-subject factors and Grade (four grade groups) as a between-subject factor. There were no significant Grade × Opponent × Block (*p* = 0.807) or Grade × Opponent (*p* = 0.879) interactions, and no main effect of Block (*p* = 0.579), indicating that the basic pattern of opponent differentiation was similar across grades.

We found a significant Opponent × Block interaction, *F* (3.75, 532.48) = 2.84, *p* = 0.027, ${\eta }_{p}^{2}$ = 0.02. Children exhibited a higher cooperation rate when facing HCO rather than LCO during block 2 (*d* = −0.34, SE = 0.02, *p* = 0.02), block 3 (*d* = −0.35, SE = 0.02, *p* = 0.018), block 4 (*d* = −0.42, *SE* = 0.02, *p* < 0.001), and block 5 (*d* = −0.53, SE = 0.02, *p* < 0.001), as opposed to block 1 (*p* = 1), This indicates that all children were able to distinguish between the two opponents after repeated interaction.

We also found a significant Grade × Block interaction, *F* (12, 568) = 2.55, *p* = 0.003, ${\eta }_{p}^{2}$ = 0.05. Children of grades 1–2 exhibited lower cooperation rates than grades 4–5 in block 3 (*d* = −0.89, *SE* = 0.04, *p* < 0.001), block 4 (*d* = −0.99, *SE* = 0.04, *p* < 0.001) and block 5 (*d* = −0.89, *SE* = 0.04, *p* < 0.001), and they also exhibited lower cooperation rates than grades 7–9 in block 4 (*d* = −0.79, SE = 0.04, *p* = 0.003) and block 5 (*d* = −0.98, SE = 0.04, *p* < 0.001). But no grade differences in block 1 and block 2, and no block differences across grades.

There were significant main effects of Opponent *F* (1, 568) = 32.39, *p* < 0.001, ${\eta }_{p}^{2}$ = 0.18, and Grade *F* (3, 142) = 10.19, *p* < 0.001, ${\eta }_{p}^{2}$ = 0.17. Participants exhibited a higher cooperation rate when facing HCO compared to LCO, *d* = −0.35, SE = 0.01, *p* < 0.001, and participants in grades 1–2 exhibited lower cooperation rates than those in grades 4–5 (*d* = −0.81, SE = 0.03, *p* < 0.001), grades 7–9 (*d* = −0.67, SE = 0.03, *p* < 0.001) and grades 10–12 (*d* = −0.46, SE = 0.03, *p* = 0.037).

### Increasing consideration of opponents’ beliefs in social learning among school-age children (Study 2)

Computational modeling in Study 2 indicated developmental differences in the relative support for reward-based and belief-based learning accounts across grade groups. All models showed good convergence, with $\hat{R}$ values below 1.1 for all parameters. Full model-comparison results, including LOOIC values, uncertainty estimates, and diagnostics, are reported in Supplementary Tables S2–S5. As in Study 1, models that included both learning and preference terms outperformed both the no-learning model and the preference-only baseline, indicating that trial-by-trial learning dynamics contributed explanatory value beyond static choice tendencies. We therefore evaluated relative support for competing model families within each grade group using LOO-based comparisons (Fig. 1a), stacking-based summaries, and posterior predictive checks (PPC). PSIS-LOO diagnostics were not uniformly ideal across all models and grade groups, particularly for the more complex variants. We therefore interpret the model-comparison results cautiously, as indicating relative differences in predictive support rather than definitive evidence of categorical model-family dominance.

For grades 1–2, the reward-based family showed greater relative support, with M1-3 providing the strongest fit within that family (LOOIC = 4520.28). PPC indicated that this model reproduced observed cooperation patterns reasonably well (${r}_{{LCO}}$ = 0.69, ${r}_{{HCO}}$ = 0.68). In contrast, for grades 4–5 and grades 7–9, the belief-based family received greater relative support, with M2-3 showing the best predictive performance within that family (grades 4–5: LOOIC = 4604.05, ${r}_{{LCO}}$ = 0.74, ${r}_{{HCO}}$ = 0.75; grades 7–9: LOOIC = 5223.59, ${r}_{{LCO}}$ = 0.70, ${r}_{{HCO}}$ = 0.75). For grades 10–12, the evidence was more mixed, although M2-3 yielded a slightly lower (LOOIC = 3829.76, ${r}_{{LCO}}$ = 0.60, ${r}_{{HCO}}$ = 0.80), the difference from the corresponding reward-based model was modest, and model weights indicated non-decisive support. We therefore interpret this oldest group as showing only partial support for belief-based accounts rather than a categorical shift in model-family dominance. M3 achieved a relatively good fit in some groups, but its additional complexity did not yield sufficient improvement in out-of-sample predictive performance to justify the extra parameters. Model recovery analyses further supported the distinguishability of the major model families (Fig. 1b). Overall, these results suggest that younger school-age children were more consistent with reward-based updating, whereas from grades 4–5 onward, there was increasing relative support for models that track opponent-specific behavioral regularities.

We then compared hyperparameters at the group level based on the best-fitting model (Tabel S6). First, we compared the two learning rates between HCO and LCO in different grades (Fig. 2a). The Bayesian Wilcoxon Signed-Rank Test showed significant differences between ${\alpha }_{h}$ and ${\alpha }_{l}$ in all grade groups. Specifically, children in grades 1–2 had higher learning rates when facing LCO than HCO (BF_10_ > 100, *W* = 7.08 × 10^6^; HCO: *M* = 0.05, SD = 0.10, 95% HDI = [0.4 × 10^−6^, 0.234]; LCO: *M* = 0.09, SD = 0.03, 95% HDI = [0.026, 0.149]), so did children in grades 10–12 (BF_10_ > 100, W = 5.31 × 10^6^; LCO: *M* = 0.012, SD = 0.02, 95% HDI = [0.2 × 10^−4^, 0.043]; HCO: *M* = 0.010, SD = 0.02, 95% HDI = [0.3 × 10^−5^, 0.044]). However, children in grades 4–5 (BF_10_ > 100, *W* = 1.06 × 10^6^; HCO: *M* = 0.015, SD = 0.02, 95% HDI = [0.2 × 10^−5^, 0.050]; LCO: *M* = 0.009, *SD* = 0.03, 95% HDI = [0.4 × 10^−8^, 0.040]) and grades 7–9 (BF_10_ > 100, *W* = 7.82 × 10^5^; HCO: *M* = 0.019, SD = 0.02, 95% HDI = [0.7 × 10^−5^, 0.072]; LCO: *M* = 0.008, SD = 0.02, 95% HDI = [0.5 × 10^−9^, 0.032]) had higher learning rates when facing HCO than LCO.

Next, a Bayesian Mann–Whitney U Test was used to compare the learning rates across different grades, excluding grades 1–2 due to the different winning model (Fig. S3 in Supplementary Materials). We found that learning rates for LCO decreased initially and then increased with age. Specifically, learning rates were significantly higher in grades 4–5 than in grades 7–9 (BF_10_ > 100, *W* = 7.07 × 10^6^), and the learning rates in grades 10–12 were significantly higher than those in both grades 7–9 (BF_10_ > 100, *W* = 4.21 × 10^6^) and grades 4–5 (BF_10_ > 100, *W* = 3.38 × 10^6^). For HCO, learning rates also exhibited a non-monotonic trend, peaking in grades 7–9. The rates in grades 7–9 were significantly higher than those in both grades 4–5 (BF₁₀ > 100, *W* = 7.15 × 10⁶) and grades 10–12 (BF₁₀ > 100, *W* = 1.07 × 10⁷), while rates in grades 4–5 were also higher than in grades 10–12 (BF₁₀ > 100, *W* = 1.00 × 10⁷).

The preference and temperature parameters were also estimated. Children in grades 1–2 had a negative value of preference (*M* = −0.57, SD = 0.11, 95% HDI = [−0.787, −0.358]), significantly lower than 0, BF_−0_ > 100, *W* = 7.50 × 10^6^, indicating that children had preferences for *Go straight* (Fig. 2b). So did children in grades 7–9 (*M* = −0.05, SD = 0.11, BF_−0_ > 100, *W* = 2.12 × 10^6^, 95% HDI = [−0.255, 0.201]) and grades 10–12 (*M* = –0.18, SD = 0.13, BF_−0_ > 100, *W* = 2.26 × 10^5^, 95% HDI = [−0.435, 0.065]). However, children in grades 4–5 had a positive preference for *Swerve*, significantly higher than 0 (*M* = 0.06, SD = 0.12, BF_+0_ > 100, *W* = 6.27 × 10^6^, 95% HDI = [−0.182, 0.284]). The value of $\theta$ in grades 4–5 was higher than grades 7–9 (BF_10_ > 100) and grades 10–12 (BF_10_ > 100), and $\theta$ in grades 7–9 was higher than grades 10–12 (BF_10_ > 100). The temperature parameter indicated a higher level of exploration and more random choice in grades 1–2 (*M* = 0.80, SD = 0.76, 95% HDI = [0.004, 1.841]). While older children had reduced exploration. A Bayesian Mann–Whitney U Test showed that temperature in grades 4–5 is lower than grades 7–9 (BF_10_ > 100, *W* = 6.86 × 10^6^) and grades 10–12 (BF_10_ > 100, *W* = 6.48 × 10^6^), and temperature in grades 7–9 is lower than grades 10–12 (BF_10_ > 100, *W* = 7.48 × 10^6^).

Finally, at the individual level, a linear regression showed that $\theta$ could also predict the averaged cooperation rates, same as Study 1, *B* = 0.24, *R*^*2*^ = 0.96, *p* < 0.001, 95% CI = [0.239, 0.254]. Then, we respectively compared BAS and BIS scores across different grades with one-way ANOVA, and found a significant main effect of grades for BAS score (*F* (3, 137) = 8.00, *p* < 0.001, ${\eta }_{p}^{2}$ = 0.15) but not for BIS score (*p* = 0.075). Specifically, BAS score in grades 1–2 is higher than grades 4–5 (*d* = 1.01, SE = 0.42, *p* < 0.001) and grades 7–9 (*d* = 0.92, SE = 0.41, *p* < 0.001). Moreover, a multiple linear regression (*θ*~Grade + BAS) controlling for grade showed that while grade positively predicted *θ* (*β* = 0.11, *p* = 0.024), the BAS score remained a significant negative predictor (*β* = −0.07, *p* = 0.016) (Fig. 2c). This indicates that stronger reward sensitivity was associated with a greater tendency to choose *Go straight*, particularly in younger children.

## Discussion

The importance of strategic social interaction lies in accurately choosing to move forward or backward at a crucial moment by observing the other’s actions. Here, we conducted two studies that manipulated behavioral tendencies of opponents (HCO vs. LCO) in a child-friendly Chicken game, applying a reinforcement learning framework to investigate social learning from preschoolers to adolescence. Our findings suggest that children across all ages adjusted their choices based on their opponents’ actions, *Swerve* (cooperate) with HCO (more aggressive) and *Go straight* (compete) with LCO (more submissive). This opponent-sensitive adaptation across development provides evidence that children rely on trial-by-trial learning rather than fixed strategies. Notably, there were age-related differences in cooperation: children aged 6–8 years displayed lower cooperation rates compared to other age groups, while children aged 8–12 years exhibited the highest cooperation rates, which then declined as they got older. Computational modeling results suggested age-related differences in the relative support for reward-based versus belief-based accounts.

We first compared behavioral performance of social learning across a wide age range (5–17 years). Consistent with previous research^44,47^, even children as young as five were able to adapt their behaviors based on different opponents. Initially, cooperation rates did not differ between the two opponents, but through repeated choices and learning, children gradually adjusted their behaviors. Interestingly, preschoolers (5- to 6-year-olds) in Study 1 demonstrated better differentiation between opponents than early primary school children (6- to 8-year-olds) in Study 2, who tended to choose *Go straight*. A plausible explanation is that the simplified payoff matrix for preschoolers reduced computational demands, whereas 6–8-year-olds faced a full matrix that included losses, which may have increased task difficulty. Nonetheless, 6- to 8-year-olds still exhibited distinct cooperation rates with two opponents, confirming the effectiveness of our experimental manipulation. As children grew older, their ability to adjust their behavior became more pronounced. Children aged 9 to 12 displayed the highest cooperation rates, aligning with the development of prosocial behavior and increased peer cooperation^39^. This age period has been identified as a “prosocial window” in developmental work, during which children show heightened sensitivity to others’ behavior and social norms. However, cooperation decreased during adolescence (12–17 years). Although this decline was not statistically significant in behavioral performance, analysis of the model parameter $\theta$, which reflects behavioral preference for *Swerve*, showed a clearly declining trend. This pattern is consistent with prior evidence that adolescents’ social decisions are strongly shaped by peer influence, potentially reducing cooperative tendencies after receiving negative/antisocial feedback^54^. Furthermore, we observed differences in BAS score across age groups, with 6–8 years exhibiting higher BAS scores than both 9–12 years and 12–15 years. Because BAS reflects motivation to seek rewards, elevated BAS in younger children may partly explain their increased use of *Go straight* responses, as competitive actions provide the highest immediate payoff in this task. We also found a negative correlation between BAS scores and $\theta$ values, with a higher BAS score being associated with more *Go straight* choices. Together, these behavioral findings demonstrate that cooperation across development is shaped jointly by learning, reward sensitivity, and age-related shifts in social motivation.

The advantages of our adjusted experimental tasks, which manipulated opponents’ behaviors in repeated social interactions, allowed us to explore the social learning process in greater depth and examine children’s decision-making flexibility. Beyond behavioral performance, computational modeling enabled us to investigate the computational mechanisms underlying strategic social interactions, providing insights into whether children’s learning processes were primarily reward-based or belief-based. To achieve this, we constructed three main learning models (reward-based, belief-based and second-order belief-based learning) alongside a no-learning model across five age groups. Moreover, each learning model included three sub-models with varying parameters: one with a single learning rate, one with two separate learning rates, and one incorporating a preference parameter. Consistent with our hypothesis, younger children (5- to 6-year-olds in Study 1 and 6- to 8-year-olds in Study 2) were more consistent with reward-based learning, whereas older children (9- to 17-year-olds) showed increasing relative support for belief-based accounts.

The reward-based model formalizes choices as being updated primarily from experienced outcomes, similar to non-social learning. Children update prediction errors, which are the differences between the expected value of a choice and the actual reward received. If the outcome exceeds expectations, its value increases, reinforcing the choice. If it falls short, the value decreases, discouraging repetition. This process enables children to adjust behavior purely based on past rewards without considering social cues or opponents’ intentions. However, more sophisticated forms of social learning may involve tracking others’ action tendencies and, in some contexts, their underlying mental states. In line with this, our results suggest that older children (9–17 years) were better captured by the belief-based model, which assumes they track opponents’ behaviors and predict future actions^15^. This model also follows RL principles, but updates based on prediction errors, which is the difference between the expected probability of an opponent’s behavior and their actual choice. Using these probabilities, participants assign subjective values to different options, refining their strategies based on tracked opponent regularities rather than current reward alone. Together, these patterns are consistent with developmental differences in the relative weighting of outcome-based versus opponent-tracking information, with children relying primarily on outcomes earlier in development and gradually placing great weight on tracking opponents’ regularities. During model comparison, although the belief-based learning model was selected as the relatively best-fitting model, the advantage over the reward-based learning model was modest. Notably, we observed comparable weights for reward-based and belief-based learning in grades 4–5 and grades 10–12. This pattern suggests that children may place increasing weight on others’ beliefs in their learning around 9–12 years. At the same time, the continued reliance on outcome information in grades 10–12 indicates that the development of social learning is not a simple linear shift toward belief-based reasoning, but rather reflects a dynamic trade-off between outcome-based and belief-based information. We also tested a second-order belief model, which builds on the belief-based model by additionally tracking opponents’ second-order beliefs when updating the expected probability of their behavior. While this model has been used in adult strategic learning research^15,16^, it did not fit the current data well in children. This suggests that both children and adolescents do not actively infer second-order beliefs in the current task.

Future studies should further explore the developmental trace of second-order belief inference. Regardless of whether learning was reward-based or belief-based, the third sub-model with a preference parameter ($\theta$) best fit the data across all age groups. The preference parameter reflects participants’ tendency to Swerve, and our results showed that it varied across ages, indicating developmental changes in behavioral preferences in social learning. Importantly, the preference should not be read as a mere “Swerve bias”, it captures a participant-specific cooperative orientation that predicts overall cooperation levels and is tightly linked to the choice of cooperating versus competing across trials. For this reason, we treat it as a summary of strategic preference rather than a single psychological construct. Taken together, our approach provides evidence for developmental changes in different computations underlying social learning, suggesting that older children increasingly track opponents’ action tendencies during social learning.

In addition to learning strategy, we observed persistent biases in learning rates based on the winning model for each age group. In Study 1, 5- to 6-year-olds exhibited higher learning rates for HCO than for LCO. According to the payoff matrix, choosing *Swerve* is a stable choice with a fixed reward, regardless of the opponent’s actions, making it easier for participants to learn HCO. In contrast, 6–8-years in Study 2, still relying on reward-based learning, showed higher learning rates for LCO than for HCO. This could be due to the changed payoff matrix, where the negative value was less well understood, encouraging more frequent *Go straight* choices. However, in older age groups, learning became more influenced by opponents’ actions. We found that 9- to 15-year-olds were more sensitive to HCO, while 15- to 17-year-olds showed greater sensitivity to LCO. Future research may further explore how social learning sensitivity to opponents develops across different ages. These findings suggest that strategic learning follows an age-related trajectory of adaptation and refinement with distinct sensitivities to specific opponents. Because task variants were age-appropriate (simplified vs. full payoff matrix), we refrain from strong cross-study causal claims and emphasize within-study patterns.

Overall, this study provides a developmental account of how children learn to coordinate and compete through reinforcement learning computations. Our findings demonstrate that children’s social learning evolves over development, adapting to opponents with different strategies and becoming increasingly sophisticated. Across age groups, model comparison suggested that younger children showed greater support for reward-based accounts, whereas older children showed greater relative support for models that track opponents’ action tendencies. In the current task, success was not merely about choosing to *Swerve* or *Go straight*, but about recognizing and acting appropriately based on the social partner. Our findings underscore that the maturation of social learning is not linear but involves the emergence of new computational strategies that support flexible, context-sensitive social behavior. This contributes to a deeper understanding of how children acquire the capacity to navigate complex social dilemmas.

It is also important to note that the present findings are based on a specific strategic interaction paradigm. The developmental patterns observed here may depend on the structure of the task and payoff matrix. Future work using alternative paradigms will be important to further test the generality of these learning patterns.

## Methods

### Participants

In Study 1, we recruited 59 healthy children from the senior kindergarten in China, 7 children were excluded due to technical malfunction, resulting in a final sample of 52 children (5.27-6.41 years old, *M*_age_ = 5.88, SD = 0.34, 26 girls). Children provided verbal assent, and written informed consent was obtained from parents or guardians prior to participation. All children received stickers as compensation. G*Power showed that this sample size gave us 97% power to detect a medium-sized effect (*f* = 0.25).

In Study 2, we recruited 148 healthy Chinese children and adolescents from primary school, middle school and high school. Two children were excluded due to technical malfunction, yielding a final sample of 146 individuals divided into four grade groups: Grades 1–2 (*N* = 39, 6.64- to 8.6-year-olds, *M*_age_ = 7.7, SD = 0.65, 15 girls) and grades 4–5 (*N* = 36, 9.38- to 12.67-year-olds, *M*_age_ = 11.08, SD = 0.75, 19 girls) in primary school, grades 7–9 in middle school (*N* = 41, 12.8- to 15.43-year-olds, *M*_age_ = 13.95, SD = 0.64, 28 girls) and grades 10-12 in high school (*N* = 30, 15.27- to 17.04-year-olds, *M*_age_ = 16.1, SD = 0.47, 17 girls). Assent and parental consent procedures were identical to Study 1. This sample size gave us 99% power to detect a medium-size effect (*f* = 0.25).

### Chicken game

The task was a child-friendly adaptation of the Chicken game, in which two players controlled cars approaching an intersection and chose either Swerve (S) or Go straight (G).

**Fig. 3:**
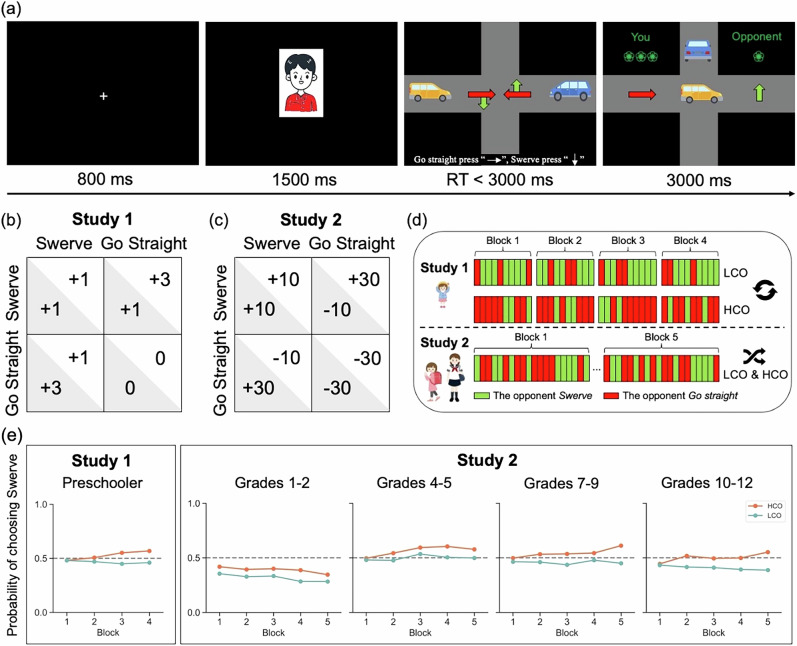
Experimental design and behavioral evidence. **a** An example of the experimental procedure in Study 1. In each trial, participants were required to choose either *Swerve* or *Go straight* within 3 s when an intersection was displayed. Feedback was then provided based on payoff matrix. In this example, the participant chose *Go straight* while the opponent chose *Swerve*, resulting in the participant receiving three flowers and the opponent receiving one flower. **b**, **c** show the payoff matrices for Study 1 and Study 2, respectively. **d** The experimental design for Study 1 and Study 2. In Study 1, preschoolers faced HCO and LCO in a counterbalanced sequence across participants, with trials divided into four blocks. In Study 2, older children from primary, middle and high school interacted with HCO and LCO mixed within each block, and participants completed five blocks of trials. **e** Participant learning performance across blocks. Participants learned to adjust their behavioral strategies across blocks when interacting with different opponents. All children gradually exhibited distinct tendencies across blocks, showing higher rates of *Swerve* when facing HCO and lower rates of *Swerve* when facing LCO.

Study 1 used a simplified payoff matrix (Fig. 3b) following a prior study^44^. Instead of using scores, the reward feedback was presented as small red flowers. The payoff structure statisfied GS > SS = SG > GG (Fig. S2). Participants had the chance to earn a better reward by choosing *Go straight* (e.g., GS), but they risked getting nothing if the opponent also chose *Go straight* (e.g., GG). Swerve, at the same time, was a safer option regardless of the opponent’s choice. Participants were told they were playing an online game with other children (the opponent) who shared their age and gender. In reality, opponents’ choices were pre-programmed and presented using E-Prime 2.0. The experiment manipulated two different opponents: a high-competitive opponent (HCO), who chose *Go straight* with a 70% probability, and a low-competitive opponent (LCO), who chose *Go straight* with a 30% probability, following prior social learning research^4,32^. Participants interacted with both opponents in separate blocks, with block order counterbalanced across participants (half encountered HCO first, half LCO first). Each participant interacted with both opponents across four blocks of 10 trials, totally 80 trials. Each trial began with an 800 ms fixation, followed by a 1500 ms display of the opponent’s image. Participants and opponents had 3000 ms to make their decisions, after which feedback was displayed for 3000 ms, with green indicating flower rewards and red indicating no payoff (Fig. 3a). Children were told that those who earned more flowers would receive more stickers. The position and color of the participants’ cars were counterbalanced, and to enhance realism, participants were asked to select an avatar to represent themselves, while their opponents chose their own avatars (Fig. S1).

In Study 2, the payoff matrix in the current study was a general version as shown in Fig. 3c. Thus, the reward outcomes were structured so that GS > SS > SG > GG. If both players chose *Go straight* (GG), they were both punished, e.g., a crash cost 30 points. In the current task, participants also interacted with two opponents (HCO & LCO), and the opponent on each trial was determined randomly. To ensure that participants learned about each opponent well, each opponent was presented in 50 trials, for a total of 100 trials. Participants were informed that those who earned more points would receive more money at the end.

### Procedure

In Study 1, children were introduced into a quiet and well-lit art classroom in the kindergarten to complete the experiment. After the participants were instructed on the rules, they were asked to answer a few questions to ensure they fully understood the tasks (e.g., How many flowers do you each get if you *Go straight* and the opponent *Swerve*?). If a child answered incorrectly, the instructions were repeated to ensure understanding before proceeding to the main task. All children completed eight training trials before proceeding to the Chicken game. Following the experimental task, detection questions with each opponent were administered to assess whether participants could distinguish between the two opponents. Participants were presented with the opponent’s avatar and asked “Which action did you observe more frequently: *Go straight* or *Swerve*?”. A total of 26 children (50%) correctly answered both detection questions, which is higher than the random level (25%), and only 4 answered neither. Finally, participants completed the *Children Neuropsychological and Behavior Scale* (CNBS), which was primarily measured using the standardized test toolbox. The CNBS is a diagnostic assessment tool widely used in China to evaluate the developmental level of children aged 0 to 6 years. It has demonstrated adequate reliability^55^. The mean score of the general Developmental Quotient (DQ) and the five subscale quotients—including gross motor, fine motor, adaptive behavior, personal-social, and language—were calculated. A DQ or subscale quotient below 70 points indicates a developmental delay. None of the children’s DQ or the five subscale quotients were less than 70 (*M* = 105.19, SD = 7.05)

In Study 2, the experimental procedure was similar to that of Study 1. A quiet classroom was selected for participants to complete the experiment. After the rules were explained and the training trials were completed, participants were asked to engage in the Chicken game. As in Study 1, children completed post-task comprehension questions assessing explicit awareness of the opponents’ patterns: In grades 1–2, 32 children (≈82%) answered both questions correctly (4 answered neither); In grades 4–5, 24 children ( ≈ 67%) answered both correctly (10 neither); In grades 7–9, 30 children ( ≈ 73%) answered both correctly (9 neither); In grades 10–12, 21 children (70%) answered both correctly (6 neither). Following the experimental task, participants completed the *Behavioral Inhibition/Activation System Scale* (BIS/BAS). The revised Chinese version of the BIS/BAS scales was used to measure the likelihood of approaching rewards (BAS) or avoiding punishments (BIS). Carver and White (1994) developed the original 20-item BIS/BAS scales^56^, which were later revised into a Chinese version^57^, with two items deleted: “Even if something bad is about to happen to me, I rarely experience fear or nervousness” and “I have very few fears compared to my friends.” The revised scales are self-report questionnaires consisting of 18 items (from 1 = strongly disagree to 4 = strongly agree). The scale measures two systems: (1) the Behavioral Activation System (BAS), with 13 items divided into three subscales—Reward Responsiveness, Drive, and Fun Seeking, and (2) the Behavioral Inhibition System (BIS), with 5 items. Higher scores indicate stronger reactivity in the respective system. The questionnaire was administered with read-aloud support for children in grades 1–2, while those in higher grades completed it via self-report. Comprehension of all items was verified before response. Cronbach’s α was calculated for each grade group: grades 1–2 (BAS: 0.73, BIS: 0.66), grades 4–5 (BAS: 0.73, BIS: 0.66), grades 7–9 (BAS: 0.70, BIS: 0.43), and grades 10–12 (BAS: 0.80, BIS: 0.85). Internal consistency was acceptable for BAS across grade groups. BIS reliability was acceptable in most groups but low in grades 7–9.

### Statistical analysis

Data preprocessing, analysis, and visualization were performed using Python 3.11.5 and R 4.4.1. Statistical analyses were then conducted using JASP 0.19 to provide Bayes factors, *p*-values, and effect sizes. For behavioral analyses, we used ANOVAs with appropriate Greenhouse–Geisser corrections when sphericity was violated. For parameter-level analyses, we used Bayesian Wilcoxon signed-rank tests and Bayesian Mann–Whitney U tests to compare learning rates and other parameters within and across groups, and Bayesian one-sample tests for comparisons against 0.

### Computational modeling

We built computational models using a hierarchical Bayesian approach, fitted in RStan with RStudio, which allowed us to generate hyperparameters at the group level to minimize error, as well as individual-level parameters. First, we developed a non-learning model (M0) that set the learning rate to 0, with only the temperature parameter $\beta \in [\mathrm{0,10}]$. Furthermore, we fitted three different models based on reinforcement learning rules, each with different hypotheses.

M1: Reward-based model. This model assumed that participants learn the value of actions from past rewards using a simple Rescorla–Wagner updating rule. The subjective value ${V}_{t}$ of each action is updated on trial *t* based on the received reward ${R}_{t}$. The difference in subjective value (*EV*) between the two options is then computed and passed through a sigmoid function to determine the probability of choosing *Swerve*. $\beta \in [\mathrm{0,10}]$ is the temperature parameter. Three sub-models were considered to fit our behavioral data. M1-1 assumed a unique learning rate $\alpha \in [\mathrm{0,1}]$, irrespective of the opponents they faced. M1-2 posited the existence of two learning rates ${\alpha }_{h}$ and ${\alpha }_{l}$, specifically attributed to different opponents.

1 $${V}_{t+1}={V}_{t}+\alpha ({R}_{t}-{V}_{t})$$

2 $${EV}={V}^{{Swerve}}-{V}^{{Go}\,{Straight}}$$

3 $$P({Swerve})=\frac{1}{1+{e}^{-\beta * {EV}}}$$

Based on M1-2, M1-3 added parameter $\theta \sim {Norm}(\mathrm{0,1})$ to capture the preference of *Swerve* option (Eq. 4).

4 $$P({Swerve})=\frac{1}{1+{e}^{-(\beta * {EV}+\theta )}}$$

M2: Belief-based model. In this model^15,16,35^, we assumed that participants tracked the opponent’s probability ${p}_{t}$ (with an initial $p=0.5$) of choosing *Swerve*. ${O}_{t}$ is the observed action of opponents, equal to 1 if the opponent chose *Swerve* and equal to 0 if the opponent chose *Go Straight*. ${V}_{t}$ of each action is derived from the payoff matrix. Similar to M1, there are three sub-models embodying distinct assumptions. M2-1 assumed a unique learning rate, M2-2 assumed two distinct learning rates ${\alpha }_{h}$ and ${\alpha }_{l}$, M2-3 assumed a preference for different options.

5 $${p}_{t+1}={p}_{t}+\alpha ({O}_{t}-{p}_{t})$$

6 $${V}^{{Swerve}}={p}_{t}\times {SS}+(1-{p}_{t})\times {SG}$$

7 $${V}^{{Go}\,{Straight}}={p}_{t}\times {GS}+(1-{p}_{t})\times {GG}$$

8 $${EV}=1-3{p}_{t}$$

M3: Second-order belief model. This model posits that the decision of participants is a function of both their and opponents’ historical choices based on M2 to track the second-order belief of opponents^13^, which was captured by parameter $\kappa \in [0,1]$. And ${Q}_{t}$ is the action of participants, ${q}_{t}$ is the opponent’s inferred probability that participants will *Swerve*. There are also three sub-models embodying distinct assumptions. M3-1 assumed a unique learning rate and kappa, M3-2 assumed two distinct learning rates ${\alpha }_{h}$, ${\alpha }_{l}$, M3-3 added a preference for different options. In Study 2, the Eq. 10 of M3 has changed due to a different payoff matrix (see details in Supplementary Materials).

9 $${p}_{t+1}={p}_{t}+\alpha ({O}_{t}-{p}_{t})+\kappa ({Q}_{t}-{q}_{t})$$

10 $${q}_{t}=\frac{1}{3}+\frac{1}{3\beta }log(\frac{1-{p}_{t}}{{p}_{t}})$$

Considering that the static preference parameter might account for a substantial portion of variance in cooperation, we performed a critical model ablation analysis. To isolate the unique contribution of the learning mechanisms, we constructed a preference‑only baseline model (M4) containing only the preference (bias) and temperature parameters, with no trial‑by‑trial updating. Two variants were specified: M4–1 (reward‑based family) and M4–2 (belief‑based family). Formal comparison between these baseline models and the full learning models confirmed that the learning computations provided significant incremental explanatory power beyond the static preference (Supplementary Materials S1–S5).

In hierarchical Bayesian modeling, the individual-level parameter *ϕ* was drawn from a group-level normal distribution *ϕ* ~ *N*(*μ*_*ϕ*_*, σ*_*ϕ*_), where *μ*_*ϕ*_ and *σ*_*ϕ*_ represent the group-level mean and standard deviation, respectively. Weakly informative priors were assigned to the group-level parameters *μ*_*ϕ*_ ~ *N*(0, 1), with its standard deviation adjusted according to relevant free parameters, and *σ*_*ϕ*_~*HC*(0, 3), a half-Cauchy distribution, whose scale parameter was similarly varied based on free parameters. All parameters were constrained to their plausible ranges following standard regularization practices.

Stan utilized Markov Chain Monte Carlo (MCMC) sampling to approximate the posterior distributions of parameters. We fitted our models with four chains of 3000 warmup iterations and 1000 additional iterations, resulting in 4000 actual samples. MCMC convergence diagnostics were performed by visual inspection of the trace plots after warmup, and an $\hat{R}$ value less than 1.1 indicated that the MCMC had converged. Model comparison was performed using a Bayesian approach. The Leave-One-Out Information Criterion (LOOIC) was used to evaluate our models via the R package “loo”^58^. The LOOIC is measured on an information criterion scale, where lower values indicate better out-of-sample prediction accuracy. Models were first grouped into three families (M1, M2, and M3). To summarize support at the family level, we performed Bayesian Model Averaging (BMA) using the stacking method within each family and compared the resulting family-level predictive support across families. The best-supported model family for each grade group was then identified. Subsequently, the preferred model within that family was selected for further analysis.

For model validation, a Posterior Predictive Check (PPC) was conducted to assess the predictive accuracy by comparing the real choice data and simulated data derived from the estimated parameters. Specifically, Pearson correlations were computed between the observed and simulated cooperation rates at the trial level. To further ensure model validity, we conducted a model recovery analysis, simulating synthetic datasets for each model and fitting these datasets with all models. We examined the proportion of participants for each generating model that was best fit by each model, using model comparison to identify the best-fitting model for each synthetic dataset. We also performed a parameter recovery analysis for the winning model. Ground-truth parameters were repeatedly sampled from a uniform prior distribution and used to generate simulated choice data, to which the same model was then refitted to obtain posterior estimates. Across 30 iterations, the recovered posterior means showed high correlations with the true parameter values (see Supplementary Materials Fig. S5), confirming that the key parameters were well-identified.

### Ethics

The study protocol was approved by the Ethics Committee in South China Normal University (SCNU-PSY-2021-051). Both studies were conducted in accordance with the University’s *Policy for Ethical Practice*, and all ethical principles outlined in the policy were followed. Children provided verbal assent, and written informed consent was obtained from parents or legal guardians.

## Supplementary information

supplementary_materials_v2

## Data Availability

The study materials and data are available on Open Science Framework (https://osf.io/fnpcy/overview).

## Code availability

The codes are available on Open Science Framework (https://osf.io/fnpcy/overview).
