# Supplementary material for: Learning to adaptively cooperate through social interactions during childhood and adolescence
Source: NPJ Sci Learn. 2026 Apr 4;11:32. doi: 10.1038/s41539-026-00423-9 (PMC13230781; doi:10.1038/s41539-026-00423-9)
Supplement: Supplementary file 1 — supplementary_materials_v2 [file 41539_2026_423_MOESM1_ESM.docx]

Supplementary Materials

**1 Experimental design**

Prior to the experiment, participants selected an avatar from a set of figures to represent themselves (Figure S1). On each trial, following their decision, they were presented with one of four possible types of feedback (Figure S2).


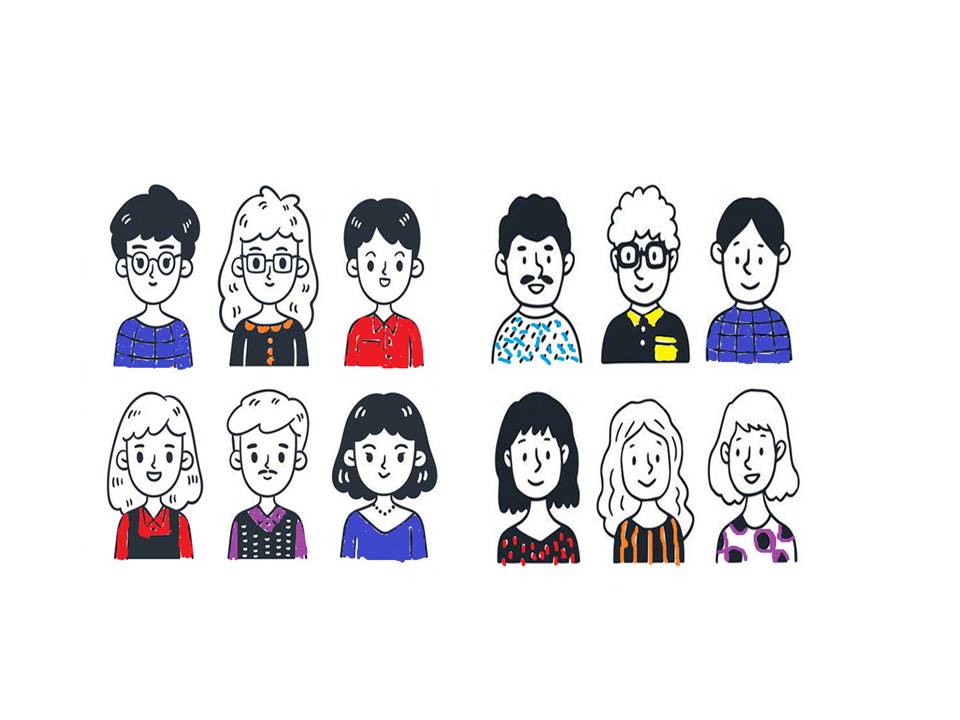


**FIGURE S1** Experimental matrials.


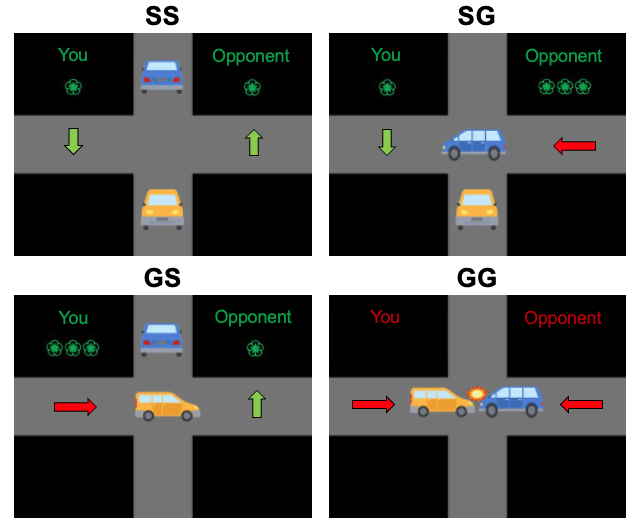


**FIGURE S2** Feedback of each trial in Study 1 (preschooler version).

**2 Computational model**

In Study 1, we did not scale the reward values for M1 and M2. For M3, which experienced convergence difficulties, the values were divided by 10.

In Study 2, we scaled the reward values for M1 and M2 by dividing them by 20. For M3, the values were divided by 60. Below are the model details for M3-3 (the second-order belief-based model) used in Study 2.

|  | $p_{t}^{Swerve}=0.5$ |
| --- | --- |
|  | $V^{Swerve}=(20p_{t}-10)/60$ |
|  | $V^{Go Straight}=(60p_{t}-30)/60$ |
| *For participant* | ${EV}^{Swerve}=(20-40p_{t})/60=\frac{1}{3}-\frac{2}{3}p_{t}^{Swerve}$ |
| *For opponent* | ${EV}^{Swerve}=(20-40q_{t})/60=\frac{1}{3}-\frac{2}{3}q_{t}^{Swerve}$ |
|  | $P(Swerve)=\frac{1}{1+e^{-\beta(\frac{1}{3}-\frac{2}{3}p_{t}^{Swerve})}}=q_{t}^{Swerve}$ |
|  | $Q(Swerve)=\frac{1}{1+e^{-\beta(\frac{1}{3}-\frac{2}{3}q_{t}^{Swerve})}}=p_{t}^{Swerve}$ |
|  | $p_{t+1}^{Swerve}=p_{t}^{Swerve}+\alpha(O_{t}-p_{t}^{Swerve})+\kappa(Q_{t}-q_{t}^{Swerve})$ |
|  | $q_{t}^{Swerve}=\frac{1}{2}+\frac{3}{2\beta}log(\frac{1-p_{t}^{Swerve}}{p_{t}^{Swerve}})$ |

**3 Model comparison and model parameters**

The LOOIC values and their standard errors (SEs) for the models in Study 1 and Study 2 are reported in Tables S1–S5. In each table, Δelpd is presented relative to the best-fitting model (M1-3 or M2-3). The proportion of Pareto k values greater than 1 is also reported. In addition, model weights were calculated within each model family. For example, in the M1 family shown in Table S1, the weights for M1-1, M1-2, and M1-3 are 0.097, 0.137, and 0.767, respectively.

| **TABLE S1** Model comparison in Study 1 | | | | | | |  |
| --- | --- | --- | --- | --- | --- | --- | --- |
| Model | Parameter | LOOIC | LOOIC_se | ∆elpd | ∆elpd_se | Pareto k > 1 | Weight |
| M0 | $\beta$ | 5007.25 | 160.01 | 221.38 | 68.64 | 25% | - |
| M1-1 | $\alpha,\beta$ | 4840.39 | 181.42 | 137.95 | 52.74 | 42% | 0.097 |
| M1-2 | $\alpha_{h},\alpha_{l},\beta$ | 4840.99 | 180.02 | 138.25 | 52.91 | 33% | 0.137 |
| **M1-3** | $\boldsymbol{\alpha}_{\boldsymbol{h}}\boldsymbol{,}\boldsymbol{\alpha}_{\boldsymbol{l}}\boldsymbol{,\beta,\theta}$ | **4564.48** | **202.53** | **0** | **0** | **25**% | **0.767** |
| M2-1 | $\alpha,\beta$ | 4966.34 | 167.65 | 200.92 | 68.53 | 37% | 0.123 |
| M2-2 | $\alpha_{h},\alpha_{l},\beta$ | 4969.33 | 166.18 | 202.42 | 68.43 | 31% | 0 |
| M2-3 | $\alpha_{h},\alpha_{l},\beta, \theta$ | 4589.54 | 197.05 | 12.52 | 10.47 | 16% | 0.877 |
| M3-1 | $\alpha,\beta$,$\kappa$ | 5158.50 | 90.95 | 310.51 | 75.68 | 64% | 0.086 |
| M3-2 | $\alpha_{h},\alpha_{l},\kappa,\beta$ | 5151.23 | 105.37 | 293.37 | 72.22 | 64% | 0 |
| M3-3 | $\alpha_{h},\alpha_{l},\kappa,\beta, \theta$ | 4643.82 | 190.51 | 39.66 | 12.96 | 17% | 0.914 |
| M4-1 | $\beta, \theta$ | 4685.94 | 190.49 | 60.73 | 18.16 | 15% | - |
| M4-2 | $\beta, \theta$ | 4685.62 | 190.52 | 60.56 | 18.39 | 25% | - |

| **TABLE S2** Model comparison in Study 2 (Grades 1-2) | | | | | | |  |
| --- | --- | --- | --- | --- | --- | --- | --- |
| Model | Parameter | LOOIC | LOOIC_se | ∆elpd | ∆elpd_se | Pareto k > 1 | Weight |
| M0 | $\beta$ | 5328.92 | 17.41 | 404.31 | 75.45 | 100% | - |
| M1-1 | $\alpha,\beta$ | 5094.24 | 81.84 | 296.73 | 69.80 | 44% | 0 |
| M1-2 | $\alpha_{h},\alpha_{l},\beta$ | 4822.02 | 106.82 | 176.52 | 41.17 | 31% | 0.10 |
| **M1-3** | $\boldsymbol{\alpha}_{\boldsymbol{h}}\boldsymbol{,}\boldsymbol{\alpha}_{\boldsymbol{l}}\boldsymbol{,\beta,\theta}$ | **4520.28** | **150.98** | **0** | **0** | **33%** | **0.90** |
| M2-1 | $\alpha,\beta$ | 5249.78 | 41.98 | 364.22 | 72.66 | 28% | 0 |
| M2-2 | $\alpha_{h},\alpha_{l}\beta$ | 4870.70 | 96.88 | 217.21 | 45.66 | 46% | 0.07 |
| M2-3 | $\alpha_{h},\alpha_{l},\beta, \theta$ | 4568.16 | 148.40 | 23.94 | 9.86 | 18% | 0.93 |
| M3-1 | $\alpha,\beta$,$\kappa$ | 5328.92 | 17.41 | 404.31 | 75.45 | 100% | 0 |
| M3-2 | $\alpha_{h},\alpha_{l},\kappa,\beta$ | 5328.92 | 17.41 | 404.31 | 75.45 | 100% | 0 |
| M3-3 | $\alpha_{h},\alpha_{l},\kappa,\beta, \theta$ | 4558.09 | 153.15 | 18.90 | 16.27 | 26% | 1 |
| M4-1 | $\beta, \theta$ | 4696.15 | 141.77 | 87.94 | 33.35 | 26% | - |
| M4-2 | $\beta, \theta$ | 4695.25 | 142.17 | 87.49 | 33.14 | 13% | - |

| **TABLE S3** Model comparison in Study 2 (Grades 4-5) | | | | | | |  |
| --- | --- | --- | --- | --- | --- | --- | --- |
| Model | Parameter | LOOIC | LOOIC_se | ∆elpd | ∆elpd_se | Pareto k > 1 | Weight |
| M0 | $\beta$ | 4958.77 | 9.14 | 177.36 | 50.03 | 100% | - |
| M1-1 | $\alpha,\beta$ | 4898.72 | 43.00 | 145.48 | 48.56 | 31% | 0 |
| M1-2 | $\alpha_{h},\alpha_{l},\beta$ | 4841.54 | 53.24 | 118.35 | 45.30 | 31% | 0.30 |
| M1-3 | $\alpha_{h},\alpha_{l},\beta,\theta$ | 4622.20 | 96.85 | 9.07 | 7.65 | 33% | 0.70 |
| M2-1 | $\alpha,\beta$ | 4888.08 | 47.00 | 144.89 | 45.54 | 33% | 0 |
| M2-2 | $\alpha_{h},\alpha_{l},\beta$ | 4773.63 | 65.19 | 102.71 | 33.14 | 47% | 0 |
| **M2-3** | $\boldsymbol{\alpha}_{\boldsymbol{h}}\boldsymbol{,}\boldsymbol{\alpha}_{\boldsymbol{l}}\boldsymbol{,\beta, \theta}$ | **4604.05** | **100.49** | **0** | **0** | **22%** | **1** |
| M3-1 | $\alpha,\beta$,$\kappa$ | 4958.77 | 9.14 | 177.36 | 50.03 | 100% | 0.01 |
| M3-2 | $\alpha_{h},\alpha_{l},\kappa,\beta$ | 4958.77 | 9.14 | 177.36 | 50.03 | 100% | 0.01 |
| M3-3 | $\alpha_{h},\alpha_{l},\kappa,\beta, \theta$ | 4628.52 | 96.67 | 12.23 | 6.06 | 25% | 0.98 |
| M4-1 | $\beta, \theta$ | 4691.24 | 92.54 | 43.59 | 26.34 | 6% | - |
| M4-2 | $\beta, \theta$ | 4697.56 | 92.20 | 46.25 | 26.31 | 11% | - |

| **TABLE S4** Model comparison in Study 2 (Grades 7-9) | | | | | | |  |
| --- | --- | --- | --- | --- | --- | --- | --- |
| Model | Parameter | LOOIC | LOOIC_se | ∆elpd | ∆elpd_se | Pareto k > 1 | Weight |
| M0 | $\beta$ | 5660.24 | 5.95 | 218.32 | 54.28 | 100% | - |
| M1-1 | $\alpha,\beta$ | 5545.48 | 67.90 | 162.04 | 42.51 | 24% | 0 |
| M1-2 | $\alpha_{h},\alpha_{l},\beta$ | 5424.99 | 83.38 | 105.84 | 31.06 | 41% | 0.07 |
| M1-3 | $\alpha_{h},\alpha_{l},\beta,\theta$ | 5281.60 | 102.29 | 29.01 | 16.54 | 24% | 0.93 |
| M2-1 | $\alpha,\beta$ | 5584.52 | 33.64 | 178.22 | 50.46 | 27% | 0 |
| M2-2 | $\alpha_{h},\alpha_{l},\beta$ | 5347.80 | 78.34 | 87.82 | 29.28 | 54% | 0.08 |
| **M2-3** | $\boldsymbol{\alpha}_{\boldsymbol{h}}\boldsymbol{,}\boldsymbol{\alpha}_{\boldsymbol{l}}\boldsymbol{,\beta, \theta}$ | **5223.59** | **107.77** | **0** | **0** | **27%** | **0.92** |
| M3-1 | $\alpha,\beta$,$\kappa$ | 5660.24 | 5.95 | 218.32 | 54.28 | 100% | 0 |
| M3-2 | $\alpha_{h},\alpha_{l},\kappa,\beta$ | 5660.24 | 5.95 | 218.32 | 54.28 | 100% | 0 |
| M3-3 | $\alpha_{h},\alpha_{l},\kappa,\beta, \theta$ | 5225.89 | 109.02 | 1.15 | 5.13 | 24% | 1 |
| M4-1 | $\beta, \theta$ | 5322.27 | 97.08 | 49.34 | 20.07 | 7% | - |
| M4-2 | $\beta, \theta$ | 5322.10 | 97.46 | 49.75 | 20.22 | 12% | - |

| **TABLE S5** Model comparison in Study 2 (Grades 10-12) | | | | | | |  |
| --- | --- | --- | --- | --- | --- | --- | --- |
| Model | Parameter | LOOIC | LOOIC_se | ∆elpd | ∆elpd_se | Pareto k > 1 | Weight |
| M0 | $\beta$ | 4131.16 | 11.68 | 150.70 | 45.43 | 100% | - |
| M1-1 | $\alpha,\beta$ | 4069.35 | 42.97 | 120.52 | 42.43 | 30% | 0 |
| M1-2 | $\alpha_{h},\alpha_{l},\beta$ | 3964.02 | 55.63 | 74.88 | 29.06 | 40% | 0 |
| M1-3 | $\alpha_{h},\alpha_{l},\beta,\theta$ | 3834.75 | 89.00 | 2.49 | 3.76 | 23% | 1 |
| M2-1 | $\alpha,\beta$ | 4057.99 | 46.59 | 116.59 | 42.16 | 33% | 0.002 |
| M2-2 | $\alpha_{h},\alpha_{l},\beta$ | 3935.00 | 60.70 | 66.47 | 28.92 | 47% | 0 |
| **M2-3** | $\boldsymbol{\alpha}_{\boldsymbol{h}}\boldsymbol{,}\boldsymbol{\alpha}_{\boldsymbol{l}}\boldsymbol{,\beta, \theta}$ | **3829.76** | **89.80** | **0** | **0** | **13%** | **0.998** |
| M3-1 | $\alpha,\beta$,$\kappa$ | 4131.16 | 11.68 | 150.70 | 45.43 | 100% | 0 |
| M3-2 | $\alpha_{h},\alpha_{l},\kappa,\beta$ | 4131.16 | 11.68 | 150.70 | 45.43 | 100% | 0 |
| M3-3 | $\alpha_{h},\alpha_{l},\kappa,\beta, \theta$ | 3832.71 | 88.95 | 1.47 | 3.98 | 13% | 1 |
| M4-1 | $\beta, \theta$ | 3911.80 | 81.74 | 41.02 | 22.93 | 13% | - |
| M4-2 | $\beta, \theta$ | 3913.06 | 81.67 | 41.65 | 22.52 | 23% | - |

The full parameter estimates for the best-supported model, along with their 95% HDIs, are presented in Table S6.

| **TABLE S6** Group-level hyperparameters for each best fitted model | | | | |
| --- | --- | --- | --- | --- |
|  | Model | Parameter | *M* (*SD*) | 95% HDI |
| Preschooler | M1-3 | $\alpha_{h}$ | 0.32 (0.11) | [0.105, 0.561] |
|  |  | $\alpha_{l}$ | 0.20 (0.15) | [0.2×10^-4^, 0.519] |
|  |  | $\beta$ | 0.37 (0.15) | [0.095, 0.687] |
|  |  | $\theta$ | 0.09 (0.19) | [-0.284, 0.476] |
| Grades 1-2 | M1-3 | $\alpha_{h}$ | 0.05 (0.10) | [0.4×10^-6^, 0.234] |
|  |  | $\alpha_{l}$ | 0.09 (0.03) | [0.026, 0.149] |
|  |  | $\beta$ | 0.80 (0.76) | [0.004, 1.841] |
|  |  | $\theta$ | -0.57 (0.11) | [-0.787, -0.358] |
| Grades 4-5 | M2-3 | $\alpha_{h}$ | 0.015 (0.02) | [0.2×10^-5^, 0.050] |
|  |  | $\alpha_{l}$ | 0.009 (0.03) | [0.4×10^-8^, 0.040] |
|  |  | $\beta$ | 2.35 (2.58) | [0.1×10^-3^, 7.969] |
|  |  | $\theta$ | 0.06 (0.12) | [-0.182, 0.284] |
| Grades 7-9 | M2-3 | $\alpha_{h}$ | 0.019 (0.02) | [0.7×10^-5^, 0.072] |
|  |  | $\alpha_{l}$ | 0.008 (0.02) | [0.5×10^-9^, 0.032] |
|  |  | $\beta$ | 2.58 (2.37) | [0.010, 7.793] |
|  |  | $\theta$ | -0.05 (0.11) | [-0.255, 0.201] |
| Grades 10-12 | M2-3 | $\alpha_{h}$ | 0.010 (0.02) | [0.3×10^-5^, 0.044] |
|  |  | $\alpha_{l}$ | 0.012 (0.02) | [0.2×10^-4^, 0.043] |
|  |  | $\beta$ | 2.91 (2.58) | [0.005, 8.307] |
|  |  | $\theta$ | -0.18 (0.13) | [-0.435, 0.065] |

Figure S3 revealed clear developmental trajectories in learning rates from grades 4-5 to grades 10-12 (shared the same model, M2-3). Learning rates of grades 1-2 were excluded from the comparison due to a different supported model (M1-3). Specifically, under the LCO condition, learning rates initially decreased before rising again in later grades. In contrast, under the HCO condition, rates increased to a peak in grades 7-9 and then decreased.


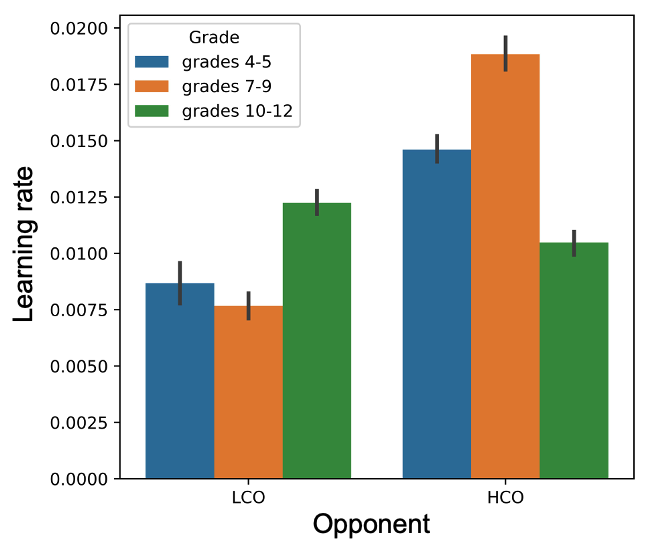


**FIGURE S3** Learning rate of grades 4-5, grades 7-9 and grades10-12 in Study 2.

**4 Model prediction**

We conducted posterior predictive check (PPC) for each winning model by using the posterior distributions of the estimated parameters to generate simulated datasets. These checks were performed at the trial level, such that the simulated and observed cooperation patterns were compared on a trial-by-trial basis rather than after averaging across trials. Figure S4 compares the simulated datasets with the original observed data, providing a visual assessment of how well the winning models captured both the overall cooperation patterns and their temporal dynamics across trials.


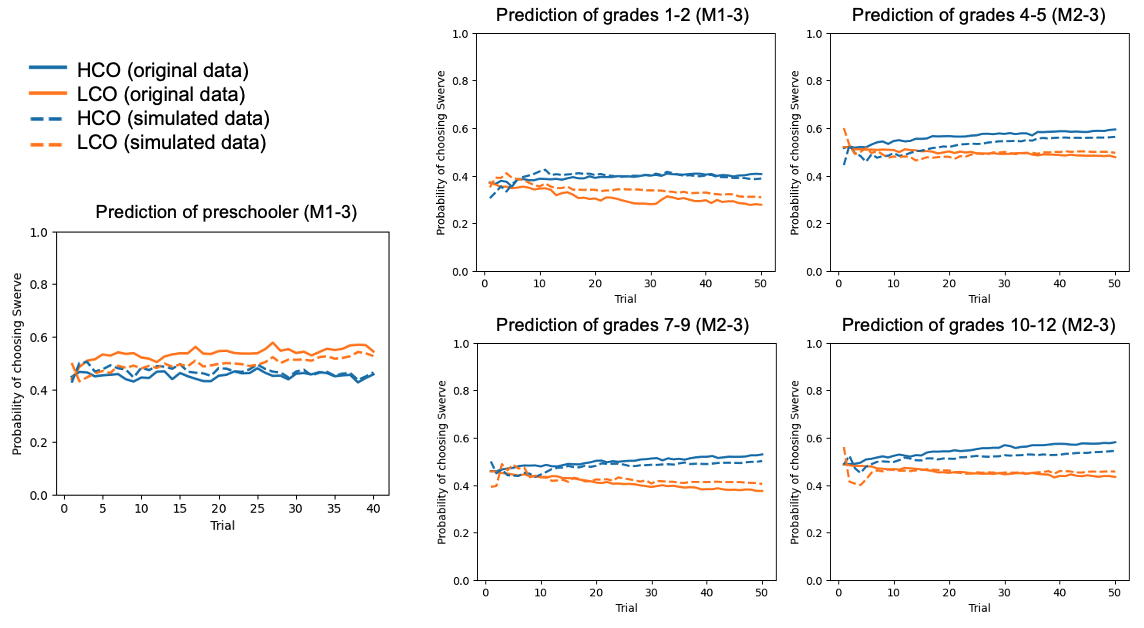


**FIGURE S4** Prediction of the winning model.

**6 Parameter recovery**

We also performed parameter recovery for each winning model using simulated datasets matched to the trial structure of the corresponding study, following the procedure described in the main text. Specifically, parameters were sampled from the prespecified prior ranges ($\alpha_{h},\alpha_{l}\in\left[ 0,1 \right], \beta\in\left[ 0,10 \right], \theta\in\left[ -5,5 \right]$), used to generate synthetic choice data, and then re-estimated using the same hierarchical Bayesian fitting procedure.

As shown in Figure S5, parameter recovery showed strong correlations for the same parameters across all age groups (preschoolers: r > 0.77; grades 1–2: r > 0.73; grades 4–5: r > 0.73; grades 7–9: r > 0.74; grades 10–12: r > 0.55). In contrast, correlations between different parameters were much lower (preschoolers: r < 0.34; grades 1–2: r < 0.45; grades 4–5: r < 0.41; grades 7–9: r < 0.23; grades 10–12: r < 0.25). These results suggest that the key parameters of the winning models were reasonably well identified and interpretable in the present design.


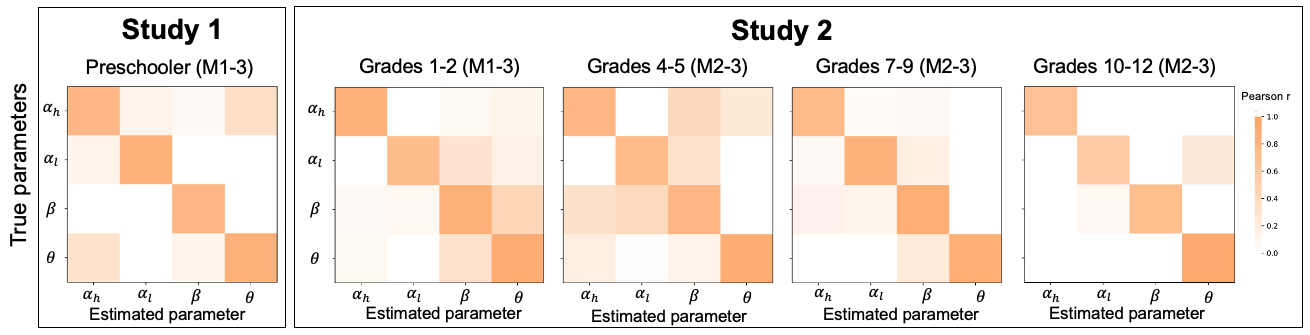


**FIGURE S5** Parameter recovery of the winning model.

**7 Robustness checks**

**7.1 Robustness to explicit opponent awareness in Study 1**

In Study 1, we conducted an additional awareness-stratified analysis using the post-task discrimination questions. Participants were divided into those who passed both questions (*n* = 26) and those who did not (*n* = 26), while the main analyses remained based on the full sample.

In both groups, the reward-based Model 1-3 showed better support than the competing belief-based model (pass group: Model 1-3 LOOIC ± SE = 2130.99 ± 159.63; Model 2-3 = 2139.01 ± 151.92; Δelpd = 4.00, SE = 7.96; non-pass group: Model 1-3 = 2437.22 ± 120.55; Model 2-3 = 2452.07 ± 121.14; Δelpd = 7.42, SE = 5.40). These results suggest that the main Study 1 conclusion did not depend on whether children passed both explicit discrimination questions.

**7.2 Robustness to non-negative reward transformation**

We conducted a robustness analysis to control for potential confounds associated with negative rewards. Specifically, we refitted Model 1-3 for younger school-age children (Grades 1–2) after adding a constant of 30 to all reward values. This transformation shifted the original payoff matrix from a range of −30 to 30 into a fully non-negative range, while preserving the relative differences between reward values. It also aligned the reward structure with the non-negative payoff format used in Study 1.

The refitted Model 1-3 yielded a LOOIC of 4540.98 (SE = 156.44). Compared with Model 2-3 (LOOIC = 4568.16, SE = 148.39), Model 1-3 showed a lower LOOIC, with a difference in expected log predictive density (Δelpd) of 13.58 (SE = 7.92). This robustness check suggests that the preference for Model 1-3 in Grades 1–2 does not depend solely on the presence of negative outcomes.

**7.3 Robustness to sex imbalance in Study 2**

We conducted an additional robustness analysis to examine whether the imbalance in the proportion of male and female participants across some grade groups affected the main behavioral findings in Study 2. Specifically, sex was entered as an additional between-subject factor in the behavioral analysis.

The results showed that neither the main effect of sex nor its interactions with the focal task factors reached statistical significance (all ps > .10). However, including sex in the model affected one of the primary findings: the Opponent × Block interaction (*p* = 0.462), which was significant in the original analysis, was no longer significant after adjustment for sex. In contrast, the Block × Grade interaction (*p* = .003), as well as the main effects of Opponent (*p* = .003) and Grade (*p* < .001), remained significant.

These results suggest that, although we did not observe direct evidence for a reliable sex effect, variation in sex composition across grade groups may influence the robustness of part of the behavioral pattern, particularly the Opponent × Block interaction. This pattern suggests that participants continued to differentiate between opponents overall, but that the evidence for increasing opponent-specific adaptation across blocks was less robust after adjustment for sex.
